# Bleeding complications after cardiac surgery, before anticoagulation start and then with argatroban or heparin in the early postoperative setting

**DOI:** 10.1186/s13019-020-1059-8

**Published:** 2020-01-28

**Authors:** Matthias Klingele, Julia Enkel, Timo Speer, Hagen Bomberg, Lea Baerens, Hans-Joachim Schäfers

**Affiliations:** 1 Department of Internal Medicine, Nephrology and Hypertension, Saarland University Medical Centre, Homburg, Saar Germany; 2 Department of Nephrology, Hochtaunuskliniken, Zeppelinstrasse 32, 61352 Bad Homburg, Germany; 3 Department of Thoracic and Cardiovascular Surgery, Saarland University Medical Centre, Homburg, Saar Germany; 4 Department of Anaesthesiology, Intensive Care and Pain Therapy, Saarland University Medical Centre, Homburg, Saar Germany

**Keywords:** Anticoagulation, Argatroban, Bleeding complication

## Abstract

**Objectives:**

After elective cardiac surgery a postoperative anticoagulation is obligatory. With critically ill patients the conventional anticoagulation standard heparin is sometimes impossible, e.g. based on HIT II. Then, argatroban is currently a possible alternative, however, due to its impaired metabolism in critically ill patients, anticoagulation effect is harder to anticipate, thus resulting in higher bleeding risk. Furthermore, to date no antidote is available. Hence, severe postoperative bleeding incidents under anticoagulation are commonly mono-causal attributed to the anticoagulation itself. This study concentrates on the number of well-defined postoperative bleeding incidents before any anticoagulation started, then actually under argatroban as well as compared to those under heparin (or switched from heparin to argatroban).

**Material and methods:**

Retrospective study including 215 patients undergoing elective cardiac surgery with a postoperative stay in ICU ≥48 h. Postoperative bleeding complications before and after start of anticoagulation were evaluated. Definition of bleeding complications were: decrease of hemoglobin by more than 2 g/dl without dilution (mean value of volume balance plus one standard deviation) and/or increased need of red blood cell transfusion/day (average transfusion rate + 2 standard deviations).

**Results:**

Within the study group of 215 patients, 143 were treated with heparin, 43 with argatroban, 29 switched from heparin to argatroban. Overall, 26.5% (57/215) postoperative bleeding complications occurred. In 54.4% (31/57) bleeding complications occurred before start of anticoagulation; in 43.6% (26/57) after. Of these, 14 bleeding incidents occurred under heparin 9.8% (14/143), 6 under argatroban 14% (6/43) and 6 switched 20.7% (6/29). Higher bleeding complications before start of anticoagulation was related to concomitant factors influencing the overall bleeding risk; e.g. score of severity of illness. These observations further correlate with postoperative, but not anticoagulation induced mortality rate of 2.8% of then given heparin, 20.9% then argatroban, 20.7% then switched.

**Conclusions:**

Postoperative bleeding complications cannot simply be attributed to anticoagulation since occurring often before anticoagulation was started. The risk for bleeding complications after start of anticoagulation was quite comparable for argatroban and heparin. Accordingly, the influence of argatroban on bleeding complications in the postoperative period may be less significant than previously thought.

## Introduction

Anticoagulation in postoperative settings is essential due to the risk of deep venous thrombosis which is higher after vascular or cardiac operations than other procedures [1]. The general probability of patients in intensive care units developing a deep vein thrombosis is estimated at 10–30% [2, 3]. Although thrombosis is rarely diagnosed after cardiac surgery (< 1%), the frequency of clinically not symptomatic thrombosis is around 20% (10–48%) despite compression therapy or aspirin in the absence of anticoagulation [4–6]. Pulmonary embolism has been described in older studies in 1–9.5% after bypass operations [4, 7]. More recent studies suggest that a higher incidence may be expected in clinically inconspicuous patients without anticoagulation (10.1–25%) [8–10]. Symptoms of embolism such as dyspnoea, thoracic pain, tachycardia, swelling of the legs, low oxygen saturation, or fever are usually associated with comorbidities or surgical trauma [10–12]. According to the German guideline (S3-AWMF), anticoagulation with unfractionated or low molecular weight heparin is recommended, resulting in significantly reduced incidences of thromboembolic events [13].

After cardiac surgery, approximately 1–5% of the patients develop heparin induced thrombocytopenia, HIT II, under heparin therapy [14–16]. In such cases, argatroban is a possible alternative for anticoagulation: argatroban is a synthetic direct thrombin inhibitor derived from L-arginine [17]. Argatroban is metabolised in the liver and excreted via the biliary system; a dose adjustment must be considered in case of hepatic dysfunction and in critically ill patients [18, 19]. The half-life of argatroban is about 45 min in healthy persons [20]. There is currently no antidote for argatroban available. Thus, concerns over an increased risk of bleeding complications under argatroban as such, as well as compared to heparin in a postoperative setting are frequently present.

Within the present study, we evaluated the actual bleeding complications under argatroban and heparin for postoperative anticoagulation during the early postoperative period after elective cardiac surgery. To put this in context, we parallel analysed factors contributing to postoperative bleeding complications as such even before start of anticoagulation.

## Subjects and methods

This was a retrospective review of a previously collected prospectively study cohort:

### Study cohort (previously collected)

From January 2010 to March 2011 all patients scheduled for elective cardiac surgery within the department of thoracic and cardiovascular surgery at the Saarland University Medical Centre were screened for inclusion. Exclusion criteria were: age < 18 years, refusal to participate, emergency admissions for cardiac surgery, haemodynamic instability necessitating emergency cardiac surgery, or inability to give written consent for participation. The study was designed as a prospective cohort study and approved by the local ethics committee (Landesärztekammer des Saarlandes; Ref. ID: 199/09). Written informed consent was obtained from all patients being included in this study. Finally, 865 patients were included [21, 22].

Clinical data were obtained during an initial standardised patient interview and subsequent review of medical documentation. Patient demographic and perioperative data were entered into a data bank.

### Actual study-group for evaluation of postoperative surgical anticoagulation

In a retrospective study design data of postoperative anticoagulation was evaluated. Based on the initial study cohort of 865 patients described above, only those with a postoperative stay in Intensive Care Unit, ICU, ≥48 h were included. Further exclusion criteria were: known endogenous factors that promote bleeding complication (e.g. von Willebrand-Jürgens syndrome); and, one patient with ruptured abdominal aortic aneurysm was excluded. Finally 215 patients were included who all received either purely heparin or argatroban or were switched from heparin to argatroban for anticoagulation in the postoperative period.

### Laboratory parameters and bleeding complications

Blood samples for biochemical monitoring were processed by the central laboratory of the Saarland University Medical Centre. Further, preoperatively liver function was assessed through the medical history screening for documented liver injury (hepatitis, alcohol, cirrhosis cardiaque, pulmonary hypertension, others). Both the Child-Pugh stage as well as the MELD score (Model for End-stage Liver Disease) on admission were calculated. A preoperative liver damage was assumed if the total bilirubin concentration was above the laboratory-specific norm combined with increased levels of GPT (Glutamat-Pyruvat-Transaminase), GOT (Glutamat-Oxalacetat-Transaminase) or GGT (Gamma-Glutamyl-Transferase), or a decreased albumin or AT III (Antithrombine) concentration. Postoperative liver cell damage was defined as an increase in GPT and/or GGT 10 times higher than the laboratory-specific norms during intensive care unit stay. In addition, every postoperative increase of the total bilirubin concentration, the maximal total bilirubin concentration and the maximal MELD score during stay in ICU were documented.

Bleeding complications were in detail defined as:

1. documented bleeding complications including increased chest tubes output and reopening for pericardial tamponade or bleeding.
2. decrease of hemoglobin by more than 2 g/dl without dilution (dilution was assumed in case of volume excess, defined as positive volume balance > than the mean value of all patients plus one standard deviation).
3. increased need for red blood cell transfusion: a transfusion occurred routinely in patients when the hemoglobin-value was below the target hemoglobin of 10 g/dl; the need was increased if the demand for erythrocyte concentrates to maintain this target hemoglobin-value exceeded two or more standard deviations of the average number of required erythrocyte concentrates by all patients.

### Anticoagulation and its monitoring

The 215 study group patients were divided into three groups according to the postoperatively given anticoagulation:

- group heparin: exclusively heparin
- group argatroban: exclusively argatroban
- group switched: switch from heparin to argatroban

The anticoagulation type and/or the switch was mostly related to the suspicion of HIT. However, the preference of the attending physician in ICU and/or surgeon also may have played a role.

Generally, various parameters were documented to assess coagulation:

1. the maximum aPTT within the first 24 h after initiation of argatroban or heparin (the target aPTT was 60-90s).
2. use of oral anticoagulants (mainly Vit-K-antagonists) or platelet aggregation inhibitors (ASS, Plavix, alone or combined) simultaneously to anticoagulation with argatroban or heparin.
3. the total number of transfused platelet concentrates (PC) and the average need of transfused PC per observation day during the intensive care stay.
4. a drop in the platelet count below 100 * 10^9^ / l.

### Statistical analysis

Continuous variables are expressed as mean ± SD (normally distributed variables) or as median and the range minimum to maximum (skewed variables). Categorical variables are presented as a percentage, unless otherwise stated. For comparisons between continuous variables the unpaired t-test was used. Categorical variables were compared by use of Chi-square test or Fisher exact test, respectively. The risk of argatroban for bleeding complications compared with heparin was initially estimated via univariate analysis. Thereafter, risk was estimated in a primary model using multivariable logistic regression including preoperative hemoglobin, pTTmax, thrombocytopenia, severity of illness (SAPS II) and surgical aspects like operation time as confounding factors. Two sensitivity analyzes were performed, such as including the confounding factors for hepatic dysfunction (Quick-value, liver cell damage, MELD max, Bilirubin max). Two-sided *p*-values < 0.05 were considered statistically significant. Data analysis was performed using IBM SPSS Statistics 23.0™ (IBM Corp., Armonk, N.Y., USA).

## Results

### Participants and descriptive data

At all, 215 patients out of the previously collected prospectively studied cohort (*n* = 865) met inclusion criteria for the retrospective evaluation of postoperative bleeding complications before start of and under anticoagulation with heparin, argatroban and switched from heparin to argatroban.

The study population was divided into three groups according to the anticoagulation: 143 patients received exclusively heparin, 43 argatroban and 29 were switched from heparin to argatroban. Baseline characteristics of study population are presented in Table 1.

**Table 1:** Baseline preoperative characteristics and comorbidities

	Study population	heparin (H)	argatroban (A)	H vs. A: *p*-value	switched (S)	H vs. S: *p*-value
Number	215	143	43		29	
	mean (SD) o. % (n)	mean (SD) o. % (n)	mean (SD) o. % (n)		mean (SD) o. % (n)	
Male	61% (131)	64% (91)	49% (21)	n.s.	66% (19)	n.s.
Age (years)	69,8 ± 10,7	68,6 ± 11,6	71,5 ± 8,4	n.s.	74,4 ± 7,2	**0,01**
BMI [kg/m2]	28 ± 4,8	28 ± 4	28 ± 5	n.s.	29 ± 7	n.s.
SAPS II Score	28,5 ± 15,3	26 ± 13	39 ± 14	**< 0,001**	27 ± 20	n.s.
EuroSCORE 2	9 ± 9,1	8,6 ± 9,1	11,1 ± 10,1	n.s.	8,0 ± 7,1	n.s.
Diabetes([%]	29% (63)	24% (35)	35% (15)	n.s.	45% (13)	**0,04**
Hb [g/dL]	13,2 ± 2,0	13,3 ± 2,0	12,5 ± 1,9	**0,02**	13,8 ± 1,6	n.s.
End stage renal desease	3% (6)	3% (5)	2% (1)	n.s.	0% (0)	n.s.
Hepatic dysfunction	5% (10)	4% (6)	2% (1)	n.s.	10% (3)	n.s.
Chronic pulmonary disease	17% (36)	15% (21)	19% (8)	n.s.	24% (7)	n.s.

Values reaching significance are merked bold

Postoperatively, there were only minor differences between the three groups regarding basic parameters. But: Patients in the argatroban and switched groups were more likely to be critically ill compared to patients receiving (only) heparin. e.g. with respect to SAPS II.

### Bleeding complications and point in time of bleeding complications

**Fig. 1:**
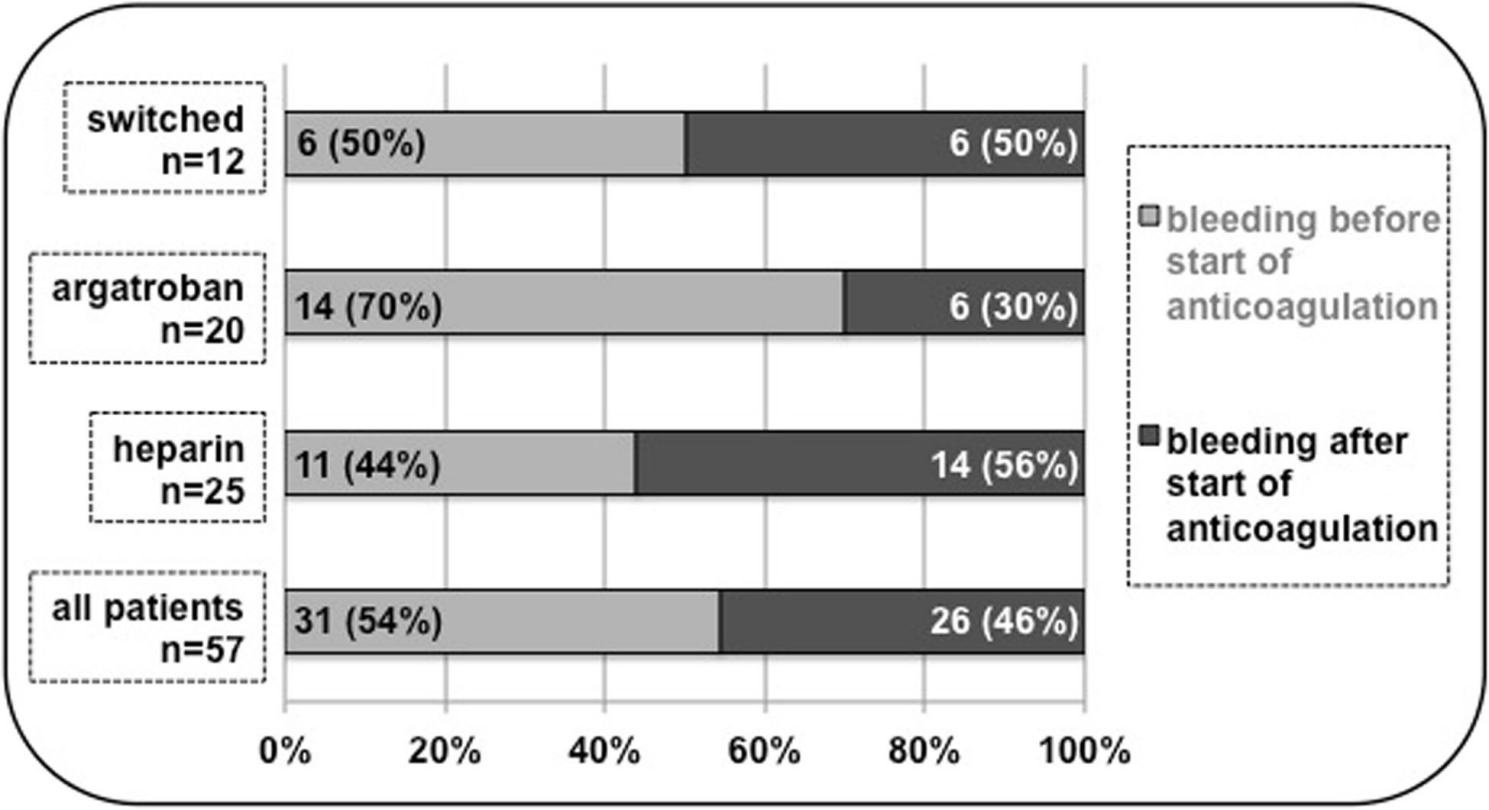
Relative occurrence of bleeding complications before and after initiation of anticoagulation per group and overall

Postoperative bleeding complications occurred in 31 (14.4%) before, 26 (12.1%) after start of anticoagulation, in total 57 (26.5%) out of 215 patients; shown in Table 2 and Fig. 1. However, incidences in patients later directly receiving or switched to argatroban were before anticoagulation start significantly, after start only minor higher compared to the exclusively heparin group.

**Table 2:** Occurrence of postoperative bleeding complications overall split into before and after start of anticoagulation, and summing up

	study population	heparin (H)	argatroban (A)	H vs. A *p*-value	switched (S)	H vs. S *p*-value
number	215 100% (215/215)	143 66.5% total (143/215)	43 20% total (43/215)		29 13.5% total (29/215)	
postoperative bleeding complication before initiation of anticoagulation	14.4% (31/215)	7.7% group (11/143)	32.5% group (14/43)		20.7% group (6/29)	
		5.1% total (11/215)	6.5% total (14/215)		2.8% total (6/215)	
postoperative bleeding complication after initiation of anticoagulation	12.1% (26/215)	9.8% group (14/143)	14.0% group (6/43)	n.s. (0.413)	20.7% group (6/29)	n.s. (0.113)
		6.5% total (14/215)	2.8% total (6/215)		2.8% total (6/215)	
postoperative bleeding complication total	26.5% (57/215)	17.5% group (25/143)	46.5% group (20/43)	**< 0.001**	41.4% group (12/29)	**0.011**
		11.6% total (25/215)	9.3% total (20/215)		5.6% total (12/215)	

Values reaching significance are merked bold

Mean period between end of surgery and start of anticoagulation was in the heparin group 6.5 h, in the argatroban 14.4 h, in the switched group 6.7 h with heparin and switched 44.4 h thereafter to argatroban. Postoperative bleeding complications before start of anticoagulation occurred within the heparin (143/215) in 7.7% (11/143), within the argatroban (43/215) 32.5% (14/43) and within the switched group (29/215) in 20.7% (6/29). Bleeding complications after start of anticoagulation occurred in 9.8% (14/143) within the heparin, in 14.0% (6/43) within the argatroban and in 20.7% (6/29) within the switched group.

An internal analysis of each group separately before and after start of anticoagulation shows indeed a relation of bleeding complications within the heparin group of 44% (11/25) before and 56% (14/25) after, within the argatroban group 70% (14/20) and 30% (6/20) after, within the switched 50% (6/12) before and 50% (6/12) after. Unfortunately, the total and each group respective number of patients with bleeding complication after initiation of anticoagulation was too small for further statistical evaluation.

### Anticoagulation and outcome

There was a clear difference in postoperative disease severity between patients who received exclusively heparin and all those who received argatroban including patients who were switched as shown in Table 3.

**Table 3:** Anticoagulation and postoperative outcome

	Study population	heparin (H)	argatroban (A)	H vs. A *p*-value	switched (S)	H vs. S *p*-value
Number	215	143	43		29	
	% (n) o. mean (range)	% (n) o. mean (range)	% (n) o. mean (range)		% (n) o. mean (range)	
Duration ICU (days)	6.3 (2–54)	4 (2–54)	9.47 (2–39)	**< 0.001**	12.83 (2–46)	**< 0.001**
Need for transfusion	83.3% (179)	74.8% (107)	100% (43)	**< 0.001**	100% (29)	**0.001**
Death	8.8% (19)	2.8% (4)	20.9% (9)	**< 0.001**	20.7% (6)	**0.002**
Due to bleeding	0% (0)	0% (0)	0% (0)	n.s.	0% (0)	n.s.
Other reasons	100%	100%	100% (43)	n.s.	100% (29)	n.s.
MELD max.	14.5 (6–40)	12.1 (6–34)	19.53 (7–40)	**< 0.001**	18.86 (8–40)	**< 0.001**
Bilirubin max.	14.5 (6–40)	1.5 (0.3–11.1)	3.0 (0.3–15.3)	**0.003**	2.9 (0.6–14.9)	**0.041**
Hepatic impairment	4.2% (9)	1.4% (2)	9.3% (4)	**0.026**	10.3% (3)	**0.034**
HIT PCR pos.	3.7% (8)	2.1% (3)	7.0% (3)	n.s.	6.9% (2)	n.s.
Need for reoperation	18.6% (40)	11.2% (16)	32.6% (14)	**0.002**	34.5% (10)	**0.003**
Due to bleeding	72.5% (29)	60% (9)	92.9% (13)	**< 0.001**	70% (7)	**0.007**
CVVHD	20% (43)	4.9% (7)	44.2% (19)	**< 0.001**	58.7% (17)	**< 0.001**

*CVVHD* continuous veno-venous hemodialysis

Values reaching significance are merked bold

Mortality in all patients was 8.8% (19/215). Mortally rate differed significantly in patients receiving heparin 2.8% (4/143) {total: 1.8% 4/215}, compared to patients receiving argatroban 20.9% (9/43) {total: 4.2% 9/215} or those switched to argatroban 20.7% (6/29) {total: 2.8% 6/215}. But: No patient died of postoperative bleeding complications.

### Parameters with impact on bleeding tendency (Table 4)

Preoperative mean platelet counts, prothrombin time, PTT-values and parameters of liver function were comparable and within normal range in all three groups. In contrast, hemoglobin was preoperatively significantly lower in the solely argatroban group compared to the heparin and switched groups. As well, signs of postoperative liver cell damage were significantly more frequent within the argatroban than the solely heparin group.

**Table 4:** Monitored parameters of anticoagulation

Number	Study population *n* = 215	heparin (H) *n* = 143	argatroban (A) *n* = 43	H vs. A *p*-value	Switched(S) *n* = 29	H vs. S *p*-value
	mean (range)	mean (range)	mean (range)		mean (range)	
PTT pre-operative [s]	30.1 (20–123)	29.2 (20–108)	33.2 (22–123)	n.s. (0.146)	29.6 (23–47)	n.s. (0.847)
PTT day 1 [s]	40.8 (23- > 140)	38.2 (23- > 140)	47.2 (29- > 140)	**0.005**	44,1 (24- > 140)	n.s. (0.229)
PTT max. within 24 h after initiation of Anticoagulation [s]	50.9 (23- > 140)	44.4 (23- > 140)	62.1 (37- > 140)	**< 0.001**	66.2 (33- > 140)	**< 0.001**
After first heparin infusion [s]	45.9 (23- > 140)	44.4 (23- > 140)	–	–	53.6 (25- > 140)	n.s. (0.073)
After first argatroban infusion [s]	61.2 (33- > 140)	–	62.1 (37- > 140)	–	59.8 (33- > 140)	B vs. C n.s. (0.663)
PTT max. Day 1–3 [s]	57.1 (28–135)	51.8 (28–135)	68.0 (38–129)	**< 0.001**	66.5 (30–119)	**0.004**
Quick preoperative [%]	868 (17–101)	89.3 (17–101)	81.4 (27–101)	**0.032**	82.3 (33–100)	**0.047**
Quick day 1 [%]	70.9 (16–101)	75.5 (28–101)	59.8 (16–98)	**< 0.001**	64.4 (22–99)	**0.001**

Values reaching significance are merked bold

### Anticoagulation and its monitoring (Table 4)

Postoperatively monitored coagulation parameters showed significantly longer PTT as well as significantly higher maximum measured PTT within the first 24 h of anticoagulation in the argatroban and the switched than in the heparin group.

### Thrombocytes

During the entire observation period, platelet counts fell to < 100*10^9^/l in 29.4% within the heparin, in 79.1% within the argatroban (*p* < 0.001), and in 69.0% within the switched group (*p* < 0.001). This reflects retrospectively the suspicion of HIT and the choice of argatroban. Postoperative platelet transfusions (after exclusion of HIT II) were more frequently within the argatroban or switched than the heparin group (60.5 and 44.8% vs. 19.6% per group; *p* < 0.001 and *p* = 0.007).

### Factors showing impact on bleeding complication

To evaluate the influence of anticoagulation (heparin and argatroban) on bleeding complications and other factors affecting coagulation, e.g. platelet count, a multivariate regression analysis was performed. Individual factors potentially influencing bleeding complication were gradually incorporated into the model. In the univariate analysis, the risk of postoperative bleeding complication as such within the argatroban-treated group is statistically higher than within the heparin-treated one (OR:4.1 *p* < 0.001). After adjustment to these former and in Table 5 described confounders the increased risk of bleeding complications before and under argatroban compared to heparin seems largely to be based on these factors (OR = 2.2, *p* = ns (0.06)). This result is also confirmed by the sensitivity analysis, with additional preoperative and postoperative factors as shown in Table 5. Of note, the influence of concomitant factors compared to all patients receiving heparin has deliberately been analysed for the argatroban group as such, independently from the point of time of start of anticoagulation.

**Table 5:** Logistic regression analysis argatroban vs. heparin (*n* = 186)

Model	Bleeding complications	
	OR (95% CI)	*p*-value
Argatroban crude	**4.1 (2.0–8.6)**	**< 0.001**
Primary model confounder before argatroban		
argatroban adjusted preoperative hemoglobin, operation time, SAPS score, pTTmax	2.2 (0.9–5.1)	0.06
Sensitivity analysis		
Preoperative confounder		
argatroban adjusted preoperative hemoglobin, quick-value, operation time, SAPS score, pTTmax	2.2 (0.9–5.2)	0.06
Postoperative confounder		
argatroban adjusted preoperative hemoglobin, operation time, SAPS score, pTTmax, liver cell damage, MELDmax, Bilimax	2.1 (0.8–5.2)	0.11

Values reaching significance are merked bold

## Discussion

Anticoagulation after actually started medication may have an impact on certain early postoperative bleeding complications in patients undergoing cardiac surgery. To the best of our knowledge, this is the first study comparing the bleeding risk of patient groups before and after start of heparin, argatroban or switched from heparin to argatroban in this vulnerable period.

In a first and rough approach, bleeding complications overall occurred more often before anticoagulation start, then indeed in a total number of the study group slightly more often in patients receiving argatroban compared to heparin, but less with switched. Nevertheless, the suspected higher bleeding risk of argatroban in the postoperative setting per se compared to heparin, since anticoagulation with argatroban is more difficult to control and to monitor for critically ill patients [19, 23], cannot be clearly confirmed.

Rather, our study reveals several aspects more likely to attribute the tendency for bleeding complications to the severity code of critical ill patients.

In this retrospective analysis, firstly, patients finally receiving argatroban were pre- and postoperatively more critically ill, had lower platelet counts and showed more disturbed coagulation parameters compared to those receiving heparin, and a significantly higher postoperative bleeding complication rate before start of anticoagulation. Thus, there is a selection bias between these groups explained by the retrospective setting and clinical decision criteria for one or another anticoagulation. This explains why in logistic regression analysis the crude bleeding risk of the argatroban group compared to the heparin one was 4.2 times higher. However, after adjusting for confounders, including start of anticoagulation, this risk was reduced formally to 2.2 times higher and was thereby no longer statistically significant.

Secondly, apart from factors impacting bleeding risk, most important was to evaluate the postoperative point in time of bleeding complication, and the point in time actually starting anticoagulation. Commonly, group affiliation is exclusively defined on the basis of anticoagulation type without the actual application status (before/ after started) in case of bleeding complication(s). But, as in clinical routine anticoagulation is not directly started after surgery, and as the time span between end of surgery and start of anticoagulation differs between patients, bleeding complications before start of anticoagulation cannot be related to any anticoagulation at all. In our study group slightly more than half of all bleeding complications occurred prior to start of anticoagulation. This extremely important aspect is only taken into account when bleeding complication group affiliation is solely based on actually started heparin and/ or argatroban treatment. Based on such sophisticated evaluation criteria, the rates of bleeding complications were comparable between the 3 groups with 9.8% heparin, 14% argatroban, and 20.7% switched under anticoagulation.

Currently, only few and inhomogeneous data of argatroban after cardiac surgery are available, confirming such rates of bleeding complication with rates of 6.3 and 12.5% with heparin and argatroban, respectively [24] and 12% with argatroban [25]. Other publications with mixed patient populations in ICUs report bleeding rates of 22% to almost 50% - with significantly more bleeding after surgical procedures than in medical patients [26, 27]. Added up, the number of included patients in each of those studies is quite low, suitable control groups are missing and often bleeding is only a secondary evaluated issue. And, those studies also had a retrospective design. Further, it shows that reported relationship between anticoagulation with argatroban and bleeding maybe questionable, as bleeding complications were often described before starting or while not applied anticoagulation, without distinguishing the yes/no status of treatment in the analysis. This is well reflected in an argatroban study of Yoon et al. with a reported bleeding rate of 64.5% after CABG surgery, but 40% bleeding incidents occurred before initiation or after argatroban application [28]. Two larger studies (304 and 418 patients) report about bleeding rates under argatroban in patients with HIT between 3% up to 11% [29, 30]; recorded up to 30 days after argatroban initiation, but the mean argatroban therapy duration was only 4–6 days.

In summary, in our study as in other studies patients are assigned to an anticoagulant group per se. But, to overcome the usual association of bleeding incidents with the respective anticoagulant simply by given group name, we deliberately distinguished whether when bleeding incidents showed anticoagulation was yet started or still/ at all applied.

### Limitations

Our study group is a negative selection of patients after cardiac surgery, only included if they stayed in ICU more than 48 h after surgery. Due to the retrospective study design, there are several biases as mentioned above, e.g. assigning patients to the different anticoagulants. The evaluated occurrence of bleeding complications during proven anticoagulation period was too small for further analysis.

## Conclusions

After cardiac surgery bleeding complications occurred significantly more often already before treatment start in those patients later then receiving argatroban versus those with heparin, or switched from heparin to argatroban. This relativly higher bleeding risk was related to concomitant factors that generally increase bleeding incident risk; e.g. severity score of illness. Additionally, more bleeding complications in total and in each sub-group as such occurred before anticoagulation start.

Evaluating the bleeding complications, our study results do not show evidence for higher bleeding risk under argatroban, however the indication of argatroban for postoperative anticoagulation should be individually and critically evaluated, especially due to the challenge of a missing antidote so far. In prospective studies evaluating postoperative anticoagulation and bleeding risk we propose to include the point in time of bleeding in relation to the start of anticoagulation, thereby the actual bleeding risk of argatroban and heparin can be further assessed.

## Data Availability

The datasets during and/or analysed during the current study available from the corresponding author on reasonable request.

## Abbreviations

AT III: Antithrombine III
GGT: Gamma-Glutamyl-Transferase
GOT: Glutamat-Oxalacetat-Transaminase
GPT: Glutamat-Pyruvat-Transaminase
HIT II: Heparin induced Thrombopenia
ICU: Intensive Care Unit
MELD: Model for End-stage Liver Disease
PTT: Partial Thrombin Time
